# Proposal of accurate cup placement procedure during total hip arthroplasty based on pelvic tilt discrepancies in the lateral position

**DOI:** 10.1038/s41598-021-93418-y

**Published:** 2021-07-06

**Authors:** Manabu Tsukamoto, Makoto Kawasaki, Hitoshi Suzuki, Teruaki Fujitani, Akinori Sakai

**Affiliations:** Department of Orthopaedic Surgery, School of Medicine, University of Occupational and Environmental Health, 1-1 Iseigaoka, Yahatanishi-ku, Kitakyushu, 807-8555 Japan

**Keywords:** Musculoskeletal system, Medical research

## Abstract

By combining the anatomical-pelvic-plane (APP) positioner with a newly improved navigation system during total hip arthroplasty (THA), it is theoretically possible to determine cup orientation based on the APP while tracking pelvic movement. The purpose of this study was to determine the navigation accuracy and whether the error is related to the pelvic position fixed by the positioner. Fifty hips that underwent primary THA between 2018 and 2020 were analysed. The accuracy was 2.34° at radiographic inclination (RI) and − 5.01° at radiographic anteversion (RA), and the error was within 10° at both RI and RA in only 40 of 50 hips (80.0%). The discrepancy in pelvic sagittal tilt was correlated with the cup orientation error and especially strongly correlated with the RA error (r = − 0.751, *p* < 0.001). When RI and RA were calculated using a correction formula to determine the true cup orientation based on the pelvic tilt discrepancies, the error in both RI and RA was within 10° in all cases (100%). The navigation accuracy is related to the pelvic position fixed by the positioner, and the correction formula for the target angle that considers pelvic tilt discrepancies can lead to accurate cup placement in the future.

## Introduction

The number of total hip arthroplasty (THA) procedures performed annually in Japan is on the rise, and in 2018, it exceeded 100,000. THA is performed in a supine or lateral body position, and more than 60% are performed in the lateral position in Japan^1^. Although the advantage of the lateral position is that we can easily place the stem and sufficiently detect impingement between the cup and stem in hip extension, the disadvantage is that it is difficult to accurately position the pelvis. In particular, discrepancies in the sagittal plane^2,3^ and intraoperative pelvic motion^4^ cause an unsuitable cup orientation.

Iwakiri et al.^5^ developed an anatomical-pelvic-plane (APP) positioner that compresses the bilateral anterior superior iliac spines (ASIS) and the pubic symphysis in the same plane in the anterior part of the pelvis and compresses the sacrum in the posterior part of the pelvis. With this device, the reference in the sagittal plane is matched with the APP by making the APP parallel to the trunk direction on the operating table. In addition, the authors created a mechanical cup navigator easily attachable to the APP positioner and demonstrated that this device helps accurately place cups^6^. They showed that accurate pelvic positioning and cup placement are possible by using an APP positioner; however, no consideration was given to intraoperative pelvic movement in their studies.

Compared with conventional methods without a navigation system in the lateral position during THA, HipAlign Lateral (OrthAlign Inc., Aliso Viejo, CA), a portable accelerometer-based navigation system (PNS), allows cups to be placed closer to the target angle by considering the trunk direction and 4 points of the acetabulum^7^. Moreover, the HipAlign New Lateral system (OrthAlign Inc., Aliso Viejo, CA), which eliminates the need for calibration of the acetabulum, has been developed in recent years, and the information required for navigation has been limited to the trunk direction.

By combining the APP positioner with the PNS, it is theoretically possible to determine the cup orientation based on the APP while tracking the movement of the pelvis during the operation. If this procedure causes a large error in the cup orientation, it is likely that the initial pelvic position determined by the APP positioner deviates. The aims of this study were to determine the accuracy of the PNS combined with the APP positioner and whether the cup orientation error is related to the position of the pelvis fixed by the APP positioner. In addition to the above purposes, if the cup orientation error and the position of the pelvis were related, we created a formula to correct the pelvic tilt discrepancies and attempted to improve the accuracy of the PNS.

## Methods

### Subjects

This study was a prospective study and approved by the Ethics Review Committee for Clinical Research of the University of Occupational and Environmental Health Institutional Review Board in accordance with the Declaration of Helsinki 2013 (approval number: H30-130). Informed consent was obtained from all participants, and all experiments were performed according to relevant guidelines and regulations.

Patients undergoing primary THA from December 2018 to April 2020 were assessed for study eligibility. The inclusion criteria were as follows: being treated by one of 4 surgeons at a single centre, having a willingness to participate, and having undergone primary THA with a single cementless acetabular component (G7: Zimmer Biomet, Warsaw, IN) and APP positioner (n = 93). The exclusion criteria included high-risk patients due to basal disease (n = 13), and specific diseases were as follows: dialysis (n = 3), severe heart failure (n = 2), history of purulent hip arthritis (n = 2), primary macroglobulinemia (n = 1), multiple myeloma (n = 1), protein C deficiency (n = 1), alcoholism (n = 1), colorectal cancer (n = 1), and common iliac aneurysm (n = 1). We do not consider that the basal disease itself affects the accuracy of PNS. Since there was a concern that the operative time would be extended due to the use of the PNS^7^, and then, at the operator's discretion, patients with underlying diseases for which the extension of the operative time could be a disadvantage were excluded from our study. The study participants signed appropriate written consent forms after the content was explained and discussed (n = 80) and underwent primary THA using HipAlign New Lateral. Since the cup orientation may change with screw use^8,9^, cases with additional screws during THAs were excluded from analysis (n = 14). In addition, cases in which the target radiographic anteversion (RA) was 20° were also excluded (n = 2). The other cases were excluded from analysis for the following reasons. Although a surgeon planned to use the PNS, the vendor forgot to prepare the PNS (n = 1). Surgeons incorrectly handled the navigation system (n = 3). Mechanical trouble with the navigation system occurred (n = 7); the breakdown of the 7 cases was sensor failure due to vibration in 2 cases, loose ball joint in 3 cases, and loose fixing pin in 2 cases. No evaluable perioperative CT due to any reason (n = 2) or cup migration (n = 1) was excluded from the analysis, and a total of 50 hips were analysed in this study.

### Surgical procedure

Fixation of the pelvis was performed with the APP positioner under general anaesthesia. Then, the film plane was held parallel to the trunk direction of the operating table (TPP; table parallel plane), and anteroposterior radiographs of the pelvis with the patient in the lateral position and the X-ray beam centred on the symphysis pubis were taken (TPP image). At that time, the operating table was tilted until the hanging chain, and the bilateral tear drops were aligned.

The navigation unit and reference sensor were paired and calibrated on a flat table. The metal pelvic base and navigation unit were percutaneously secured with two parallel 3.2-mm pins and 1 oblique pin to the ipsilateral iliac crest using sterile techniques^7^. Before an incision was made, the sagittal plane of the trunk was registered by holding the long probe parallel to the TPP (Supplementary Fig. 1). The surgical approach was the direct lateral approach. During the placement of the final acetabular component, the PNS displayed radiographic cup abduction and anteversion angles with respect to the TPP. Therefore, we assumed that the TPP and APP were made parallel by the APP positioner, and the radiographic inclination (RI) and RA angles based on the navigation record (NR) were calculated considering the pelvic inclination of the APP in the supine position (PI_APP_). We set the target cup orientation angle to 40° at RI and 15° at RA. All patients received cementless acetabular components (G7; Zimmer Biomet, Warsaw, IN), and additional screw fixation was not performed.

### Measurement of cup orientation using pre- and postoperative computed tomography (CT) images

All subjects underwent pre- and postoperative CT scans in the supine position, and postoperative CT scans were performed at 1 week after THA. Cup orientation parameters (RI and RA) were measured using ZedHip (LEXI Co., Ltd., Tokyo, Japan), and the measurement methodology described in a previous report was used^6^. Since the intraclass correlation coefficients (2, 1) for interobserver reliability of the cup orientation measurement using ZedHip for 16 subjects selected randomly were 0.971 [95% confidence interval (CI) 0.919–0.990] for RI and 0.980 (95% CI 0.945–0.993) for RA, the measurements were performed by each operator.

### Discrepancies in pelvic tilt

**Figure 1:**
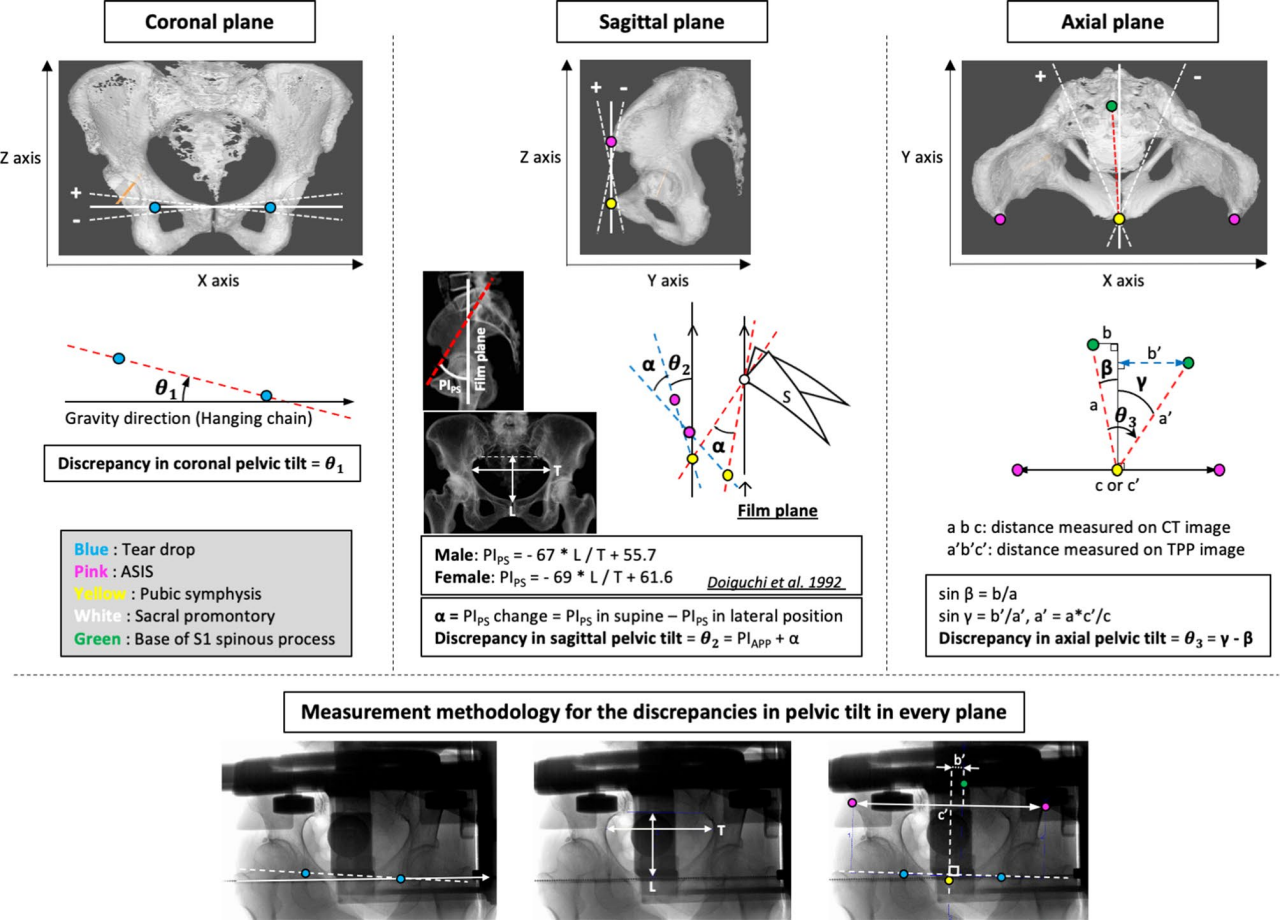
Measurement methodology for the discrepancies in pelvic tilt in every plane. When the operative side was on the right, the leftward inclination in the coronal plane, the forward tilt in the sagittal plane, and the anterior rotation in the axial plane were defined as positive. ASIS, anterior superior iliac spine; S, sacrum; PI_PS_, pelvic inclination of plane formed by pubic symphysis and sacral promontory; PI_APP,_ pelvic inclination of anatomic pelvic plane in supine position; CT, computed tomography; TPP, table parallel plane.

The measurement methodology for discrepancies in pelvic tilt in each plane are shown in Fig. 1. For the discrepancy in coronal pelvic tilt (θ_1_), the angle between the hanging chain and the line connecting the bilateral tear drops was measured using the TPP image. The discrepancy in sagittal pelvic tilt (θ_2_) was determined by the change from the pelvic inclination formed by pubic symphysis and sacral promontory (PI_PS_) in the supine position to PI_PS_ in lateral positions using CT scout view and TPP image. This procedure is currently widely used in Japan because it allows the sagittal tilt of the pelvis to be calculated from frontal X-ray images^10^. The vertical diameter of the pelvic foramen (L) divided by the horizontal diameter of the pelvic foramen (T) (L/T ratio) was measured on the anteroposterior radiographs of the pelvis, and PI_PS_ was calculated from the formula using the L/T ratio. The methods used were theoretically similar to those described by Nishihara et al.^11^ The discrepancy in axial pelvic tilt (θ_3_) was calculated using axial CT images and TPP images. In advance, axial CT images were used to determine the angle between the line connecting the pubic symphysis and the base of the S1 spinous process and the line perpendicular to the APP (β). By measuring the distance of the bilateral ASIS (c′) and the shortest distance from the base of the S1 spinous process to the perpendicular line through the pubic symphysis of the line connecting bilateral tear drops (b′) on the TPP image (γ), the discrepancy in axial pelvic tilt (θ_3_) was calculated. All radiological analyses were performed by one investigator using the software program Image VINUS Web (Yokogawa Electronics Inc., Tokyo, Japan)^12,13^. We have presented the intra-/interobserver coefficient correlations and the validation of Doiguchi’s methods in Supplementary Fig. 2. Regarding the intraobserver reliability of the pelvic tilt measurement for 16 subjects selected randomly, the intraclass correlation coefficient (1, 1) for the discrepancy in each plane was 0.967 (95% CI 0.911–0.988) for the coronal plane, 0.995 (95% CI 0.987–0.998) for the sagittal plane and 0.861 (95% CI 0.655–0.949) for the axial plane.

### Statistical analyses

The results for the continuous variables are presented as the means ± standard deviations. The Shapiro–Wilk test was used to determine whether the variables followed a standard normal distribution, and between-variable associations were analysed using the Pearson correlation coefficient. A paired t-test was used to compare the cup orientation errors before and after the correction formula was used to calculate the true cup orientation. *p* values of < 0.05 were considered statistically significant. We performed a power analysis and found that a minimum of 47 cases were required to perform simple correlation analysis (α = 0.05, power = 0.8, effect size = 0.4). All statistical analyses were performed using STATA/IC 16 (StataCorp, College Station, TX, USA).

## Results

### Postoperative cup orientation and accuracy

**Figure 2:**
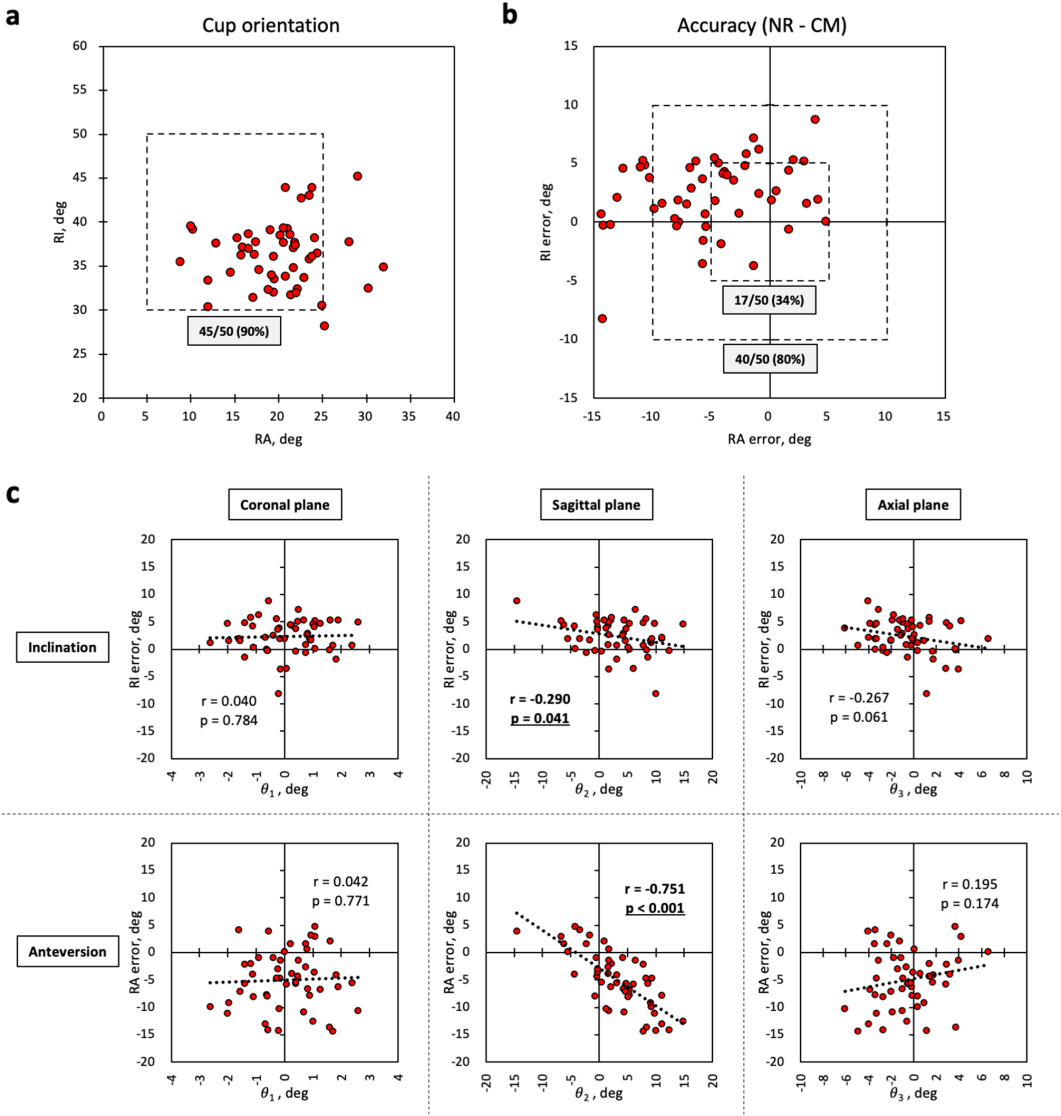
Accuracy of acetabular cup orientation, relationship between the cup orientation error and discrepancies in pelvic tilt. **a** Cup orientation, as measured by the postoperative CT images, and **b** accuracy of the portable accelerometer-based navigation system (HipAlign New Lateral Position). **c** The correlation diagram shows the relationship between RI/RA error and discrepancies in pelvic tilt in every plane. r is the correlation coefficient. The dotted square in (**a**) shows the Lewinnek safe zone. NR, navigation record; CM, CT measurement; RI, radiographic inclination; RA, radiographic anteversion; θ_1_, discrepancy in coronal pelvic tilt; θ_2_, discrepancy in sagittal pelvic tilt; θ_3_, discrepancy in axial pelvic tilt.

The demographic data are shown in Table 1. The average postoperative cup orientation value determined by the CT measurements (CMs) was 36.38° ± 3.7 at RI and 20.00° ± 5.0 at RA (Table 2), and 45 of 50 hips (90%) were in the safe zone as described by Lewinnek^14^ (Fig. 2a).

**Table 1:** Demographic characteristics of the enrolled patients.

Variable	N = 50
Age (range), years	71.0 (33–84)
Female, n (%)	44 (88.0)
Height (range), cm	154.1 (141–168)
Weight (range), kg	58.9 (42–86)
Body mass index (range), kg/m^2^	24.7 (17.2–32.4)
**Diagnosis, n (%)**	
Osteoarthritis	43 (86)
Avascular necrosis	6 (12)
Rapidly destructive coxopathy	1 (2)

**Table 2:** Perioperative CT data and navigation records (NRs).

Variable	N = 50
**Pre- and postoperative CT measurement (CM)**	
PI_APP_ measured by preop CT, deg	2.66 ± 6.6 (− 15.8 to 13.4)
RI measured by postop CT, deg	36.38 ± 3.7 (28.2–45.2)
RA measured by postop CT, deg	20.00 ± 5.0 (8.8–31.9)
**NR**	
RI predicted from the NR, deg	38.72 ± 2.9 (33.5–45.7)
RA predicted from the NR, deg	14.99 ± 4.1 (6.8–25.8)
**Accuracy (NR—CM)**	
RI error, deg	2.34 ± 3.1 (− 8.2 to 8.8)
RA error, deg	− 5.01 ± 5.2 (− 14.4 to 4.8)

The results for the continuous variables are presented as the means ± standard deviations with ranges in parentheses.

*CT* computed tomography, *op* operative, *PI*_*APP*_ pelvic inclination of anatomic pelvic plane in supine position, *RI* radiographic inclination, *RA* radiographic anteversion.

In addition, the RI and RA predicted from NR when we assumed that the TPP and APP were made parallel by the APP positioner are shown in Table 2. The average postoperative cup orientation value based on the NR was 38.72° ± 2.9 at RI and 14.99° ± 4.1 at RA. The accuracy (NR—CM) was 2.34° ± 3.1 at RI and -5.01° ± 5.2 at RA (Table 2). The error was within 5° at both RI and RA in only 17 of 50 hips (34%), and the error was within 10° at both RI and RA in only 40 of 50 hips (80%) (Fig. 2b).

### Relationship between cup orientation error and discrepancy in pelvic tilt

The discrepancy in sagittal pelvic tilt was correlated with the error in cup orientation (RI or RA) and especially strongly correlated with RA error (RI error: r = − 0.290, *p* = 0.041, RA error: r = − 0.751, *p* < 0.001) (Fig. 2c). The discrepancy in axial pelvic tilt also tended to weakly correlate with RI error (r = − 0.267, *p* = 0.061). The discrepancy in coronal pelvic tilt was not correlated with either RI error or RA error.

### Correction formula calculating true cup orientation

**Figure 3:**
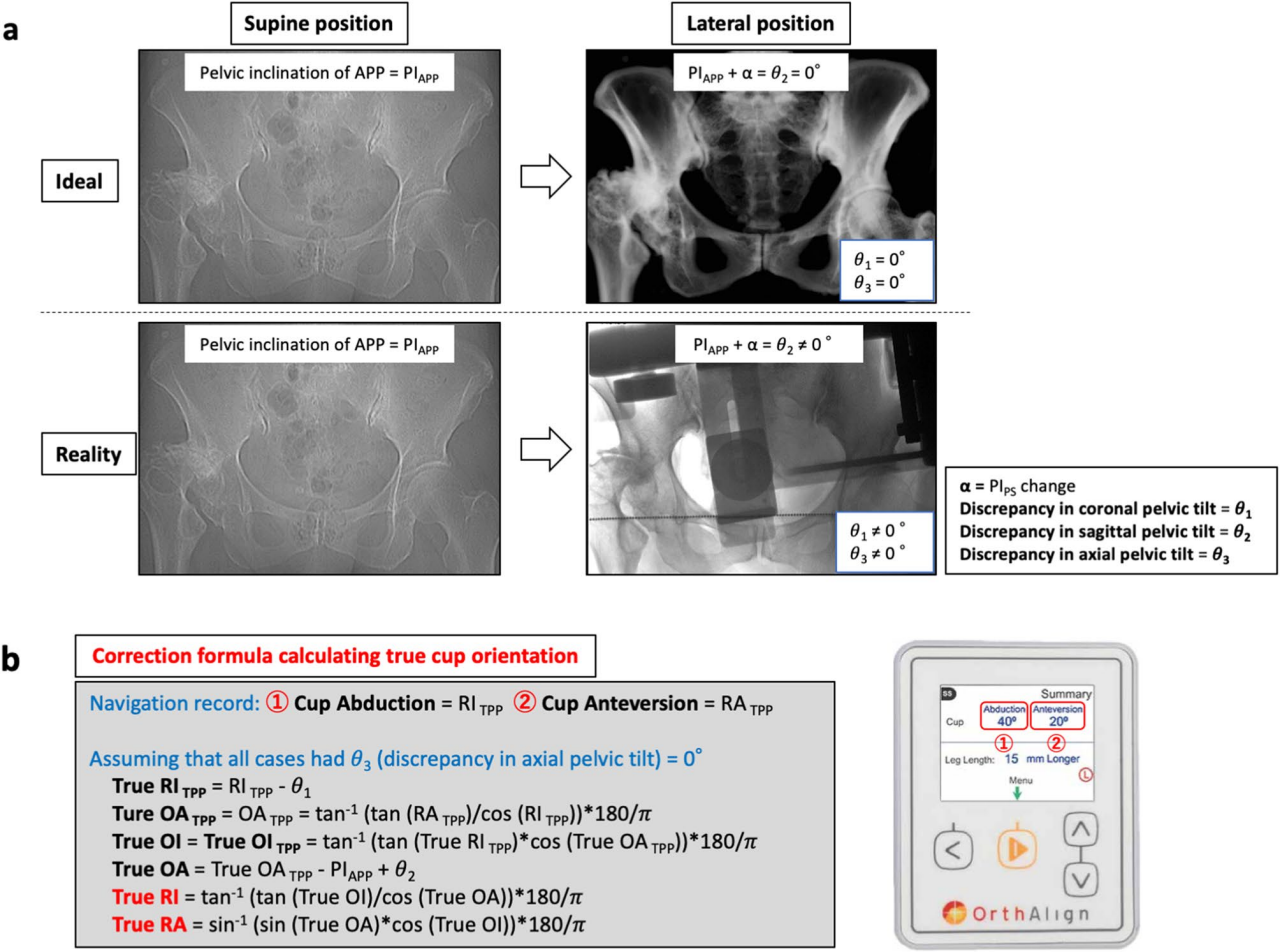
Correction formula calculating true cup orientation based on the discrepancy in pelvic tilt, as measured by TPP images. (**a**) Although the APP should ideally be parallel to the TPP, pelvic tilt discrepancies were observed in many cases in reality. (**b**) We created a correction formula to calculate the true cup orientation based on the pelvic tilt discrepancies (θ_1_ and θ_2_) measured by the TPP images. APP, anatomic pelvic plane; PI_PS_, pelvic inclination of plane formed by pubic symphysis and sacral promontory in supine or lateral position; RI, radiographic inclination; RA, radiographic anteversion; OI, operative inclination; OA, operative anteversion; TPP, table parallel plane; RI_TPP_, RI based on the TPP; RA_TPP_, RA based on the TPP; OI_TPP_, OI based on the TPP; OA_TPP_, OA based on the TPP.

**Figure 4:**
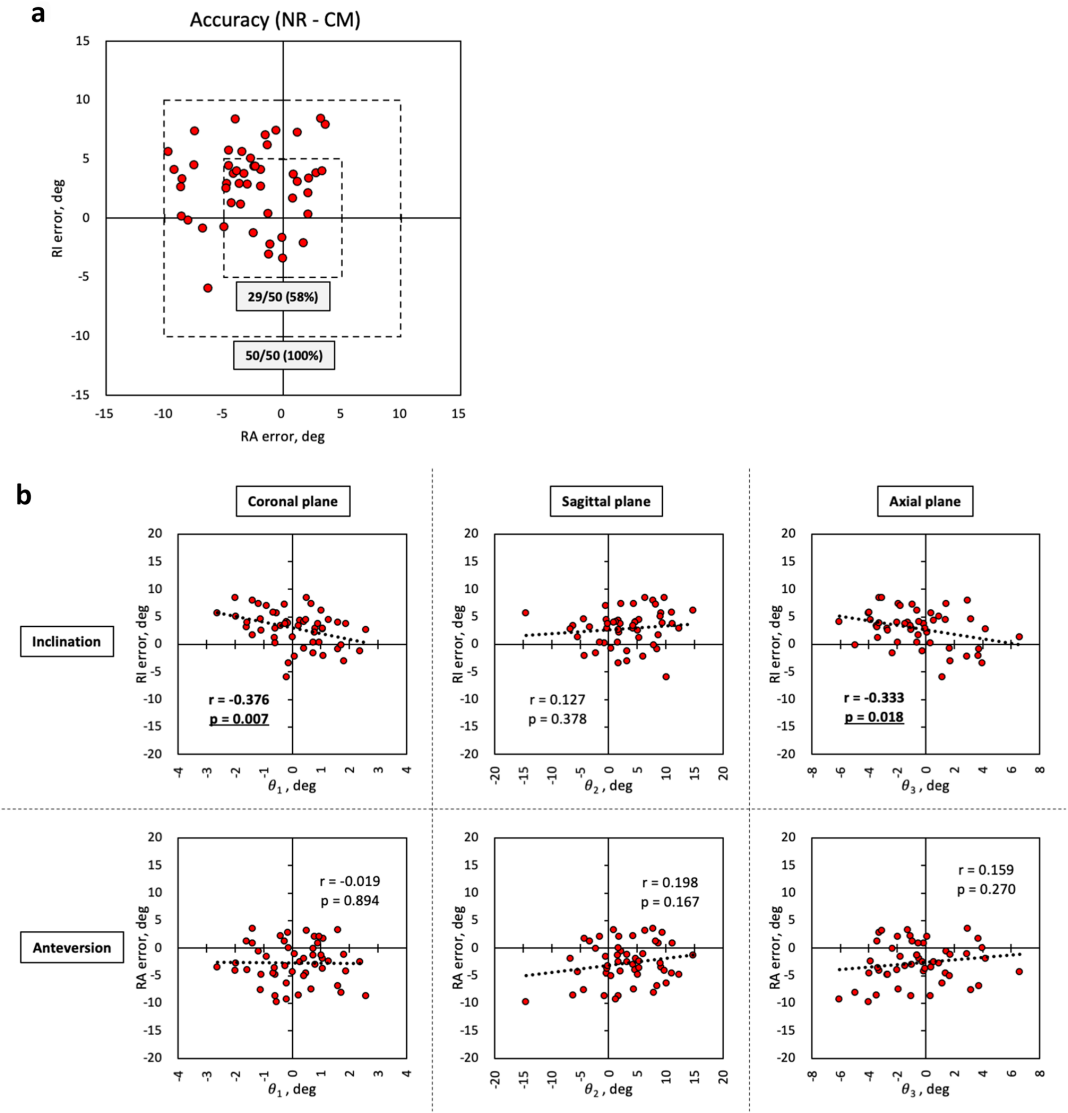
Accuracy when using the correction formula, and relationship between cup orientation error using correction formula and discrepancies in each pelvic tilt. (**a**) The actual accuracy of the portable accelerometer-based navigation system. The use of the correction formula clearly reduced the number of cases with large errors exceeding 5° or 10°. (**b**) The correlation diagram shows the relationship between the true RI/RA error and discrepancies in pelvic tilt in each plane. Since θ_1_ and θ_2_ were corrected, the correlation between the RI/RA error and θ_2_ in Fig. 2 disappeared. The uncorrected θ_3_ remained correlated with RI error. r is the correlation coefficient. NR_,_ navigation record; CM, CT measurement; RI, radiographic inclination; RA, radiographic anteversion; θ_1_, discrepancy in coronal pelvic tilt; θ_2_, discrepancy in sagittal pelvic tilt; θ_3_, discrepancy in axial pelvic tilt.

Although we aimed to place the APP parallel to the vertical axis of the operating table, in reality, discrepancies in pelvic tilt were observed in many cases (Fig. 3a). Since the value displayed by the navigation system was based on the TPP, to calculate the actual cup orientation angle based on the NR, discrepancies in the pelvic tilt for the ideal pelvis position should be considered. When actual RI and RA were calculated using the correction formula shown in Fig. 3b (assuming that all cases had θ_3_ = 0°) to determine the true cup orientation angle based on NR, the error in both RI and RA was within 5° in 29 of 50 cases (58%) (Fig. 4a). The error in both RI and RA was within 10° in all cases (100%), and the RA error with the correction formula was significantly lower than that without the correction formula (Table 3). In contrast, the RI error tended to increase when using the correction formula. Regarding the relationship between cup orientation error using the correction formula and the discrepancies in each pelvic tilt, the discrepancy in sagittal pelvic tilt no longer correlated with RI error or RA error, and the uncorrected discrepancy in axial pelvic tilt remained correlated with RI error (r = − 0.333, *p* = 0.018) (Fig. 4b).

**Table 3:** The comparison of the cup orientation errors before and after the correction formula was used.

	Before	After	*P* value
**NR—CM**			
RI error, deg	2.34 ± 3.1 (1.5–3.2)	2.89 ± 3.3 (2.0–3.8)	0.036
RA error, deg	− 5.01 ± 5.2 (− 6.5 to − 3.5)	− 2.69 ± 3.6 (− 3.7 to − 1.7)	0.001
**NR—CM (absolute value)**			
RI error, deg	3.16 ± 2.2 (2.5–3.8)	3.73 ± 2.2 (3.1–4.4)	0.021
RA error, deg	5.99 ± 4.1 (4.8–7.1)	3.69 ± 2.6 (3.0–4.4)	< 0.001

Comparisons were performed using the paired *t* test. The results for continuous variables are presented as the means ± standard deviations, with 95% confidence intervals in parentheses.

*NR* navigation record, *CM* CT measurement, *RI* radiographic inclination, *RA* radiographic anteversion.

## Discussion

We investigated the accuracy of the newly improved PNS and the relationship between the cup orientation angle determined by this device and the position of the pelvis fixed by the APP positioner. The accuracy was 2.34° at RI and − 5.01° at RA, and the error was within 10° at both RI and RA in only 40 of 50 hips (80%). The discrepancy in sagittal pelvic tilt was correlated with error of cup orientation and especially strongly correlated with RA error. Thus, we calculated the true cup orientation using the correction formula based on the discrepancy in coronal and sagittal pelvic tilt, as measured by the TPP images. When true RI and RA were calculated using the correction formula to determine the true cup orientation angle based on the NR, the errors in both RI and RA were within 10° in all cases (100%). The RA error with the correction formula was significantly smaller than that without the correction formula. The implications for this finding are described below.

Tanino et al.^7^ showed that the absolute deviations of the postoperative measured angles from the target position for cup inclination and anteversion were 3.7° ± 3.0 (range 0–13.0) and 6.0° ± 4.5 (range 0.1–19.0), respectively, in the PNS group. In addition, 51 of 55 hips (93%) in the navigation group were positioned within the Lewinnek safe zone. Based on the above results, our data demonstrated that the improved PNS, which eliminated the need for calibration of the acetabulum, is comparable to the previous PNS. First, regarding the Lewinnek safe zone, we showed that 45 of 50 hips (90%) were within the safe zone. Next, regarding the accuracy of PNS, the absolute value of RI error was 3.16 ± 2.2 (range 0–8.8), and RA error was 5.99 ± 4.1 (range 0.1–14.4). Unfortunately, however, it seemed to be inferior to the CT-based navigation system^15^.

Iwakiri et al.^5^ reported that APP positioner holds promise as a means of reducing sagittal pelvic tilt in a simple, minimally invasive, and highly cost-effective manner. However, in our study, it was extremely difficult for us to make the APP and the body trunk direction of the operating table completely parallel, even when we used the APP positioner. The average discrepancy in sagittal pelvic tilt was 3.2° ± 5.7 (range − 14.5 to 14.8), and some adjustments could be needed to correct the sagittal pelvic tilt with the APP positioner. In addition, we described the superiority of the APP positioner, but it was almost the same as the result of Tanino et al.'s study, who did not use the APP positioner. Therefore, we should discuss the effect of APP positioner on PNS and consider the factors that could not perform the placement of the acetabular component correctly. The report by Iwakiri et al., which is the developer of APP positioner, does not consider coronal tilt or intraoperative pelvic movement, but they minimized the discrepancy of sagittal pelvic tilt. In addition, some patients were excluded from the analysis to control the variances in sagittal tilt and axial tilt in their study^5,6^. The main cause of the RA error in our study is that there were cases in which the discrepancy in sagittal pelvic tilt was significantly larger than that in their study, and it is considered to be a precaution when using PNS combined with APP positioner. In addition, although there were no cases in which the coronal pelvic tilt was extremely misaligned, there were also cases in which the pubic symphysis and sacrum were significantly misaligned with respect to the axial pelvic tilt. However, despite having performed pelvic fixation using the APP positioner with more than 50 patients, we still feel it difficult to parallel the APP of the pelvis with the TPP. In addition, it wastes considerable time to place the pelvis accurately. Thus, we have considered correcting the orientation of the pelvis by using a correction formula on the TPP image taken just before surgery.

Kanazawa et al.^16^ reported various pelvic tilts and movements during THA in the lateral decubitus position, and Hasegawa et al.^17^ showed that preoperative, intraoperative, and postoperative changes in the pelvic tilt angle were risk factors for the absolute values of PNS errors in RA. In addition, Asai et al.^18^ reported that pelvic tilt reduced the accuracy of the acetabular component using PNS in an in vitro study. In our study, the improvement in accuracy using the pelvic tilt correction formula means that the accuracy of PNS depends on the pelvic tilt at the time of pelvic fixation and can be evidence that the movement of the pelvis during THA can be tracked. Asai et al.^18^ also mentioned that surgeons should use a solid pelvic lateral positioner to reduce discrepancies in pelvic tilt when using PNS in the lateral decubitus position. At least until the sagittal plane of the trunk is registered using the PNS, the pelvis needs to be firmly fixed, and we believe that the APP positioner that can firmly fix the pelvis is useful.

**Figure 5:**
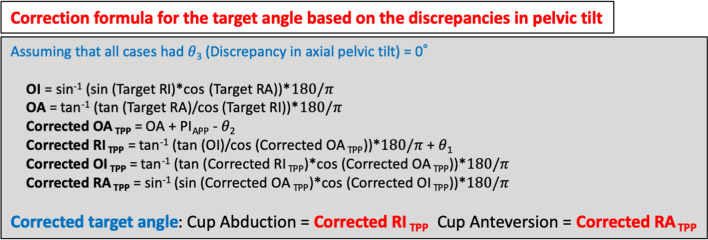
Correction formula for the target angle based on the discrepancies in pelvic tilt, as measured by TPP images. In the future, we plan to use this correction formula to determine the target cup orientation angle. It will be necessary to minimize the angle of θ_3_ in order to achieve more accurate installation. RI, radiographic inclination; RA, radiographic anteversion; OI, operative inclination; OA, operative anteversion; TPP, table parallel plane; RI_TPP_, RI based on the TPP; RA_TPP_, RA based on the TPP; OI_TPP_, OI based on the TPP; OA_TPP_, OA based one TPP; θ_1_, discrepancy in coronal pelvic tilt; θ_2_, discrepancy in sagittal pelvic tilt; θ_3_, discrepancy in axial pelvic tilt; PI_APP_, pelvic inclination of anatomic pelvic plane in the supine position.

The fact that the error in RA was reduced by correcting the deviation of the pelvis tilt on the TPP images may mean that the accuracy of the device itself is extremely good. Therefore, based on our results, we created a correction formula for target angle based on the discrepancy in coronal and sagittal pelvic tilt, as measured by the TPP images to achieve precise cup placement (Fig. 5). We consider that more accurate cup placement will be possible by calculating the discrepancies in coronal and sagittal pelvic tilt based on the TPP image taken just before the operation. In fact, if we set these formulas in files, such as Excel or Numbers, we can correct target angle simply by measuring the L/T ratio of the pelvic foramen and the angle between the hanging chain and the line connecting the bilateral tear drops (θ_1_). However, as shown in Fig. 4b, the discrepancy in axial pelvic tilt is associated with RI error even when coronal and sagittal pelvic tilt is corrected. Thus, to increase the accuracy of cup placement, the discrepancy in axial pelvic tilt needs to be as close to 0° as possible, and we are planning to conduct additional studies using the PNS with the attention to this point.

Our study had some limitations. Because it is generally believed that it is difficult to measure sagittal or axial pelvic tilt from the anteroposterior view of the pelvis^19^, concerns remain about the measurement error on TPP images. In fact, it is almost impossible to accurately measure axial pelvic tilt from the anteroposterior view of the pelvis. However, although it may also be difficult to accurately measure sagittal pelvic tilt (PI_APP_ or PI_PS_) on the anteroposterior view of the pelvis, we believe that it is relatively possible to accurately measure the angle changes in sagittal pelvic tilt between the supine and lateral positions.

Our data demonstrated that the newly improved portable accelerometer-based navigation system, which eliminates the need for calibration of the acetabulum, combined with the APP positioner is comparable to the previous portable accelerometer-based navigation system. However, discrepancies in sagittal pelvic tilt related to the error in cup orientation were observed in many patients, even when we used the APP positioner. We believe that correction for the target angle based on the discrepancies in coronal and sagittal pelvic tilt, as measured by TPP images, will lead to more precise cup placement.

## Supplementary Information

Supplementary Information 1.

Supplementary Information 2.
