# Supplementary figures and images for: Proposal of accurate cup placement procedure during total hip arthroplasty based on pelvic tilt discrepancies in the lateral position
Source: Sci Rep. 2021 Jul 6;11:13870. doi: 10.1038/s41598-021-93418-y (PMC8260668; doi:10.1038/s41598-021-93418-y)

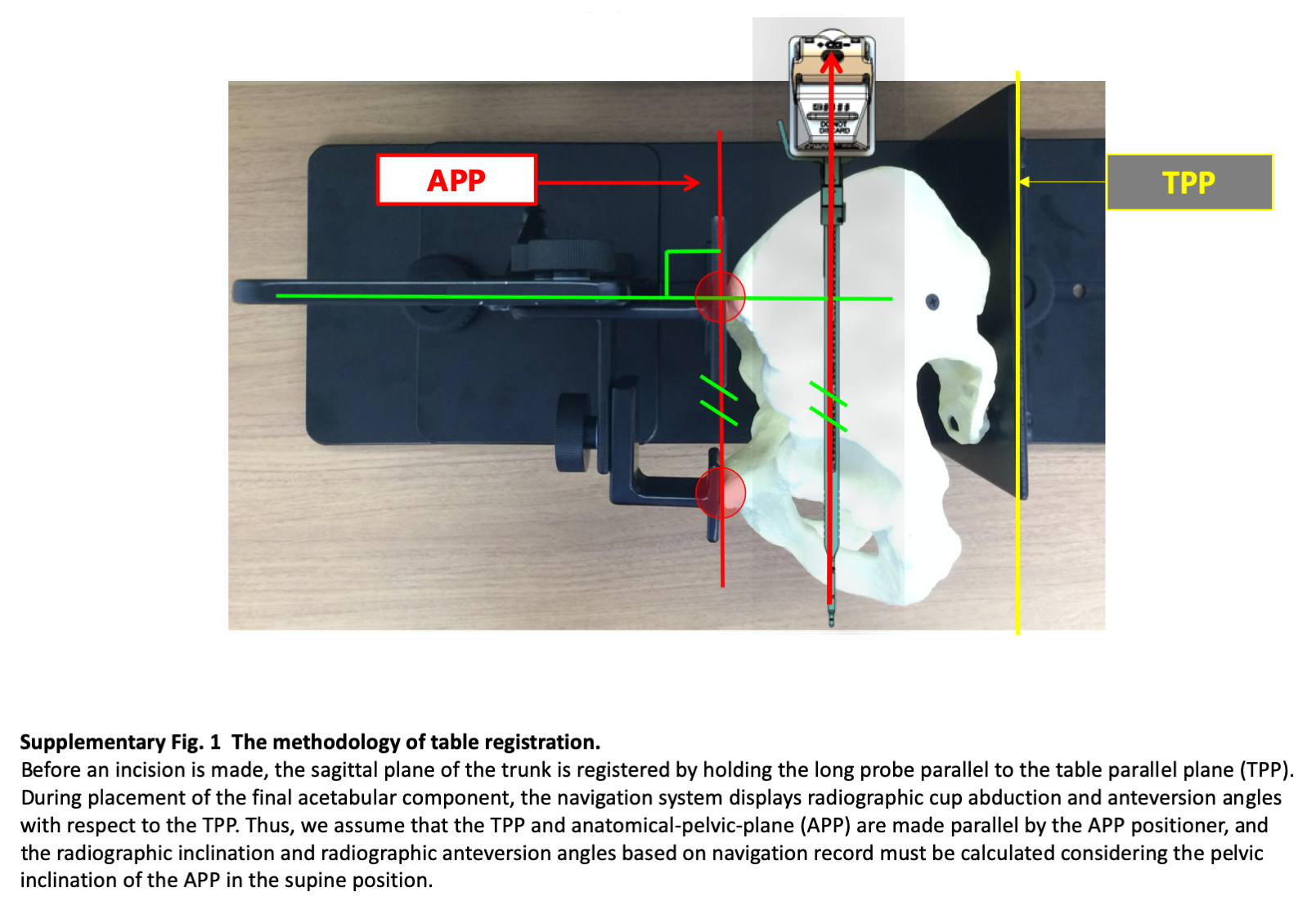

Supplement: Supplementary file 1 — Supplementary Information 1. [file 41598_2021_93418_MOESM1_ESM.tiff]

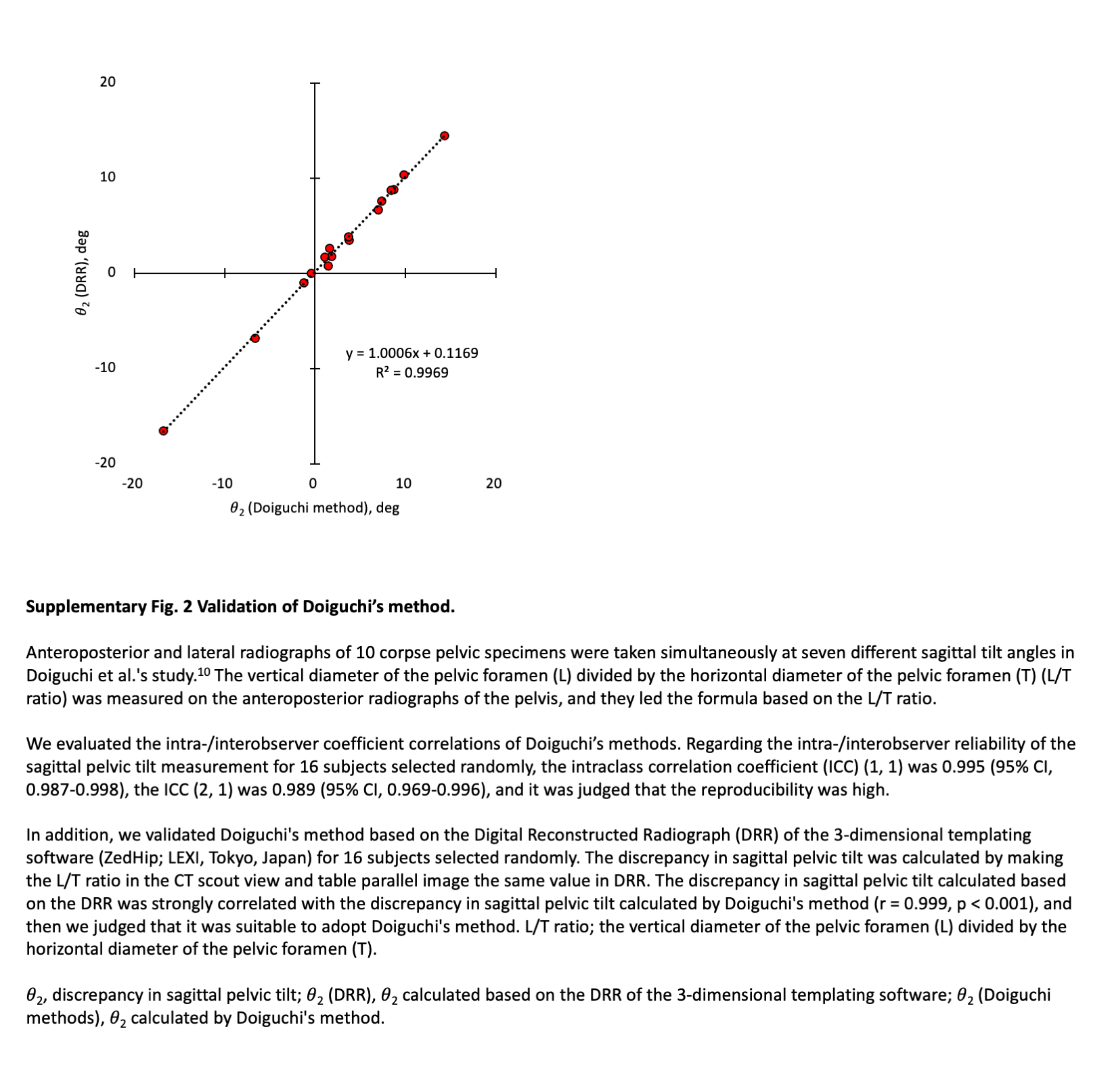

Supplement: Supplementary file 2 — Supplementary Information 2. [file 41598_2021_93418_MOESM2_ESM.tiff]
